# A Novel Variable Stiffness Torque Sensor with Adjustable Resolution

**DOI:** 10.3390/mi16080868

**Published:** 2025-07-27

**Authors:** Zhongyuan Mao, Yuanchang Zhong, Xuehui Zhao, Tengfei He, Sike Duan

**Affiliations:** School of Electrical Engineering, Chongqing University, Chongqing 400044, China; 20231101103g@stu.cqu.edu.cn (Z.M.); 20221101040@stu.cqu.edu.cn (X.Z.); 20221101087g@stu.cqu.edu.cn (T.H.); 20241101121g@stu.cqu.edu.cn (S.D.)

**Keywords:** torque sensor, adjustable resolution, adjustable range, MRF, torsion spring, screw modulation method

## Abstract

In rotating machinery, the demands for torque sensor resolution and range in various torque measurements are becoming increasingly stringent. This paper presents a novel variable stiffness torque sensor designed to meet the demands for high resolution or a large range under varying measurement conditions. Unlike traditional strain gauge-based torque sensors, this sensor combines the advantages of torsion springs and magnetorheological fluid (MRF) to achieve dynamic adjustments in both resolution and range. Specifically, the stiffness of the elastic element is adjusted by altering the shear stress of the MRF via an applied magnetic field while simultaneously harnessing the high sensitivity of the torsion spring. The stiffness model is established and validated for accuracy through finite element analysis. A screw modulation-based angle measurement method is proposed for the first time, offering high non-contact angle measurement accuracy and resolving eccentricity issues. The performance of the sensor prototype is evaluated using a self-developed power-closed torque test bench. The experimental results demonstrate that the sensor exhibits excellent linearity, hysteresis, and repeatability while effectively achieving dynamic continuous adjustment of resolution and range.

## 1. Introduction

In the field of rotating machinery, the demand for precise torque measurement is increasingly growing to achieve more accurate control [1]. Torque sensors, as a core component of measurement, play a crucial role in industrial applications, such as electric vehicles, robotics, and aerospace [2,3,4]. For instance, in factory performance evaluation, dynamic calibration tests typically involve mounting the factory torque sensor on a dedicated test rig. It is then evaluated against higher-performance torque sensors for various performance metrics, such as resolution, range, response speed, and stability, to ensure accuracy and reliability, meeting expected performance requirements [5]. However, the performance requirements of factory torque sensors vary with changing demands, necessitating adjustments in the range and resolution required for high-performance torque sensors. Nevertheless, most sensors cannot enhance both parameters simultaneously because range and resolution are inherently contradictory [6]. Generally, a larger range results in lower resolution, and vice versa. In actual dynamic calibration tests, high-performance torque sensors must be replaced according to different testing requirements. However, frequent replacements not only require multiple high-performance torque sensors but also lead to decreased accuracy, increased labor costs, and reduced efficiency due to repeated installations [7]. Therefore, to address the aforementioned issues, it is necessary to provide a novel high-performance torque sensor with adjustable resolution (TSAR) across different ranges.

Torque sensors primarily consist of sensitive elements and detection elements [8]. Sensitive elements endure torque and produce linear deformation, while detection elements measure this deformation and determine the torque by establishing a relationship between torque and deformation [9]. Traditional torque sensors are typically strain-based and offer advantages such as small size and low cost [10,11]. Power supply and signal transmission for such sensors usually require brushes or slip rings [12]. However, friction in brushes and slip rings can cause issues such as reduced lifespan and accuracy [13]. To address this issue, Chen proposed a rotational shaft torque and rotational speed measurement method coupling resistance and capacitance, which essentially solved the difficult problems in signal transmission and power supply [14]. In recent years, the investigation of rotary-type torque sensors has gained significant attention due to their characteristics of frictionless and gapless operation [15]. Distinguished from traditional strain-type torque sensors, these sensors predominantly consist of a sensitive element and a measurement unit. The transfer of torque in such sensors is achieved through the elastic deformation of the sensitive element. Subsequently, the detection of this deformation is accomplished by the measurement unit, utilizing methods such as capacitance [16], electromagnetic [17], and optical techniques [18]. The ensuing data is then further processed into torque information. Han et al. [19] proposed a high-sensitivity joint torque sensor with floating beams and supporting beams. Lai et al. [20] proposed a Fiber Bragg Grating (FBG)-based torque sensor with high sensitivity and miniature size for MIS instruments and multi-finger hands. Tiwari et al. [21] presented the design, modeling, and fabrication, as well as the experimental validation, of the multi-axis µForce-Torque sensor with a range-to-resolution ratio of 55000. Ding et al. [22] proposed a high-resolution resonant torque sensor based on a MEMS quartz resonator for the torsion test of micro-scale materials. Yeh et al. [23] proposed a contactless thin-layered high-resolution torque sensor with a fully digital signal processing circuit. Jiang et al. [24] sequentially proposed two distinct sensor designs. The first is a high-resolution instantaneous torque sensor based on the double-eccentric modulation principle. The second is a double-resolver high-sensitivity torsion spring-type torque sensor based on the iterative error self-compensation method. While the torque sensors discussed earlier demonstrate notable advantages in sensitivity and resolution, their limitations become evident in the context of measurement range, rendering them particularly suited for applications involving small torque and demanding high-precision measurements. Liu et al. [25] presented the novel parallel load-sharing principle of six-dimensional heavy force/torque, resulting in a substantial improvement in the measurement range of the sensor. Brookhuis et al. [26] proposed a miniature large range multi-axis force–torque sensor for biomechanical applications. While the aforementioned torque sensor boasts a sizable measurement range, high natural frequency, and elevated rigidity, its notable drawbacks manifest in the realm of measurement accuracy.

There are various methods for angle measurement, such as common encoders [27], sine–cosine encoders [28], and resolvers [29]. However, the aforementioned sensors are plagued by several issues. For example, encoders often have low resolution, and resolvers are susceptible to magnetic interference. In mechanical measurement, linear distance measurement technology is more mature and precise compared to angle measurement technology. Therefore, converting angle measurement to linear distance measurement is a highly effective method. Traditional methods achieve contact-based measurement through the motion relationship between cams and pushrods. Jiang et al. [13] proposed an eccentric modulation technique that uses an eddy current displacement sensor aligned with an eccentric sleeve to achieve non-contact high-precision angle measurement. However, the eccentric sleeve has eccentric inertia, which can lead to imbalance in rotating components, causing mechanical vibration and noise. This imbalance can also accelerate mechanical wear, leading to instability in the motion of rotating components, thereby reducing system accuracy and performance. Moreover, eddy current displacement sensors are sensitive to magnetic fields, making them unsuitable for the design presented in this paper. To address these issues, this paper proposes a screw modulation method, designing a sinusoidal screw and replacing the eddy current displacement sensor with a laser displacement sensor to achieve non-contact high-precision angle measurement. It should be noted that ‘non-contact’ here refers to the angle sensing method. The torque-induced rotation is captured by a laser displacement sensor without physical contact, as opposed to traditional torque sensors that might use contacting elements like strain gauges or potentiometers. The proposed torque sensor still requires a mechanical interface (shaft connection) with the test object to transmit torque, as is the case for any torque sensor. Because the laser displacement sensor measures the deformation optically, we do not need sliding contacts, bearings, or brush assemblies to transmit the signal from a rotating shaft. This not only improves the sensor’s longevity and reliability by eliminating parts that wear out due to contact, but it also reduces maintenance requirements. Another merit is the high precision and cleanliness of the signal. The laser sensor provides a high-resolution analog output without the quantization or noise issues that some encoders have.

A review of the previous works reveals the inherent contradiction between a sensor’s range and resolution. Typically, sensors can only have excellent performance on a single parameter, whether it be sensitivity, accuracy, or range. Sun et al. [30] presented a design method for a large-range torque sensor with variable resolutions based on the principle of variable stiffness. Nevertheless, the sensor can only accommodate two resolution adjustments and lacks the capability for continuous adjustability in resolution. Subsequently, Sun et al. [31] presented a novel force sensor with a variable configuration, capable of dynamically altering the measuring range and force resolution by adjusting stiffness. They also proposed the concept of a torque sensor with variable stiffness. However, this concept is difficult to realize because achieving variable stiffness by altering the mechanical structure during rotational motion is highly challenging.

To address the aforementioned issues, this study proposes a novel torque sensor. Unlike other typical torque sensors, the sensor proposed in this study uses screw modulation technology to detect the output rotation angle of an elastic element, thereby measuring the applied torque. The elastic element is a variable stiffness structure (VSS). The VSS achieves dynamic continuous adjustment from low to high stiffness by utilizing the varying shear stress properties of magnetorheological fluid (MRF), thus maintaining high resolution over a wide range. Therefore, this sensor can reduce stiffness to enhance measurement accuracy or increase stiffness to extend the measurement range, meeting different requirements for measurement accuracy and range under various conditions.

## 2. Mechanical Design

### 2.1. Operating Principle

**Figure 1:**
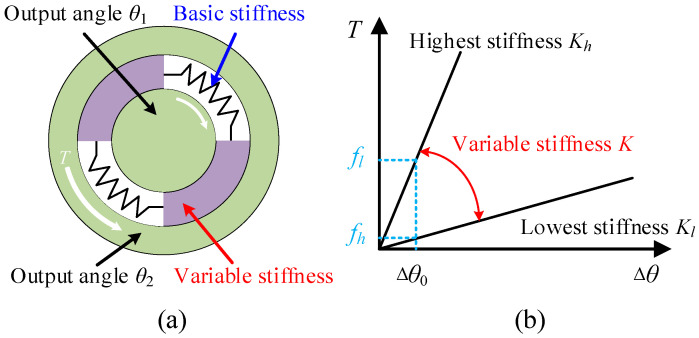
Schematic of the operating principle. (**a**) Diagram of the TSAR. (**b**) Relationship between torque and angle.

The design concept of the TSAR is illustrated in Figure 1a. The TSAR primarily consists of an outer ring, an inner ring, and a variable stiffness elastic element in between. It is assumed that the outer and inner rings are made of materials with infinitely high stiffness and do not deform under any circumstances. The elastic element has properties of linear deformation and continuously adjustable stiffness. When the outer and inner rings are subjected to equal and opposite torques, the elastic element experiences shear stress and deforms. This deformation causes different angular displacements *θ*_1_ and *θ*_2_ in the outer and inner rings, respectively, with the angular displacement difference being Δ*θ*. This angle is measured using screw modulation technology (detailed later), which offers advantages such as non-contact measurement, analog signal output, high precision, and strong anti-interference capabilities. Regardless of the stiffness variation in the elastic element, as long as the maximum shear stress on the elastic element is less than its yield shear stress, the relationship between torque and Δ*θ* will be linear, with a stiffness *K*, as shown in Figure 1b. Assuming the angular resolution of the resolver is Δ*θ*_0_, the highest and lowest resolutions of the TSAR are *f_h_* and *f_l_*, respectively. It is evident that the resolution of the TSAR is influenced by the stiffness *K*. Additionally, the yield shear stress increases with stiffness, thereby expanding the measurement range of the TSAR. Therefore, the sensor can reduce stiffness to improve measurement accuracy or increase stiffness to expand the measurement range, meeting various requirements for measurement accuracy and range under different conditions.

**Figure 2:**
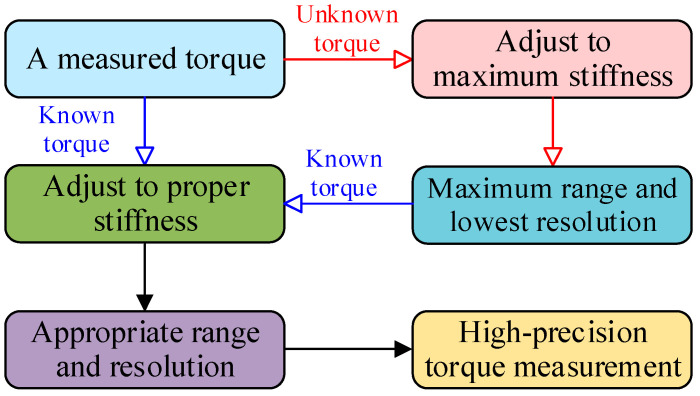
Working flow diagram of the TSAR.

The workflow of the TSAR is shown in Figure 2. In a torque sensor, all torque measurements should maximize measurement accuracy within an appropriate range. Torque measurement can be categorized into two scenarios: for known torque, the TSAR can be calibrated with a single stiffness adjustment to achieve the appropriate resolution and range. For unknown torque, calibration with lower stiffness is required to enhance accuracy. Specifically, the stiffness of the TSAR is first adjusted to the maximum; then, the maximum range and lowest resolution of the TSAR are selected for an initial torque measurement to ensure the measured torque does not exceed the sensor’s range, thereby avoiding damage. Next, by reducing the stiffness of the TSAR, the sensor achieves the highest resolution within an appropriate range, thus allowing high-precision torque measurement.

### 2.2. Design of the VSS Based on MRF

The key to achieving adjustable resolution and range for the TSAR lies in the design of the elastic element. The elastic element must meet two requirements: first, it should minimize the base stiffness to enhance sensitivity while transmitting torque with minimal loss. Second, the elastic element must be able to adjust the stiffness over a wide range and respond quickly.

However, most elastic materials exhibit low yield shear stress under low stiffness conditions, which can lead to a shorter fatigue life of the elastic element. Torsion springs, as mechanical springs, store and release energy through torsional deformation. When a torsion spring is subjected to torque, it undergoes torsional deformation, with its torsion angle following Hooke’s Law in relation to the torque. Torsion springs feature high sensitivity and high yield shear stress, meeting the requirements for high reliability and long lifespan. Therefore, torsion springs are well-suited as the elastic elements of torque sensors.

**Figure 3:**
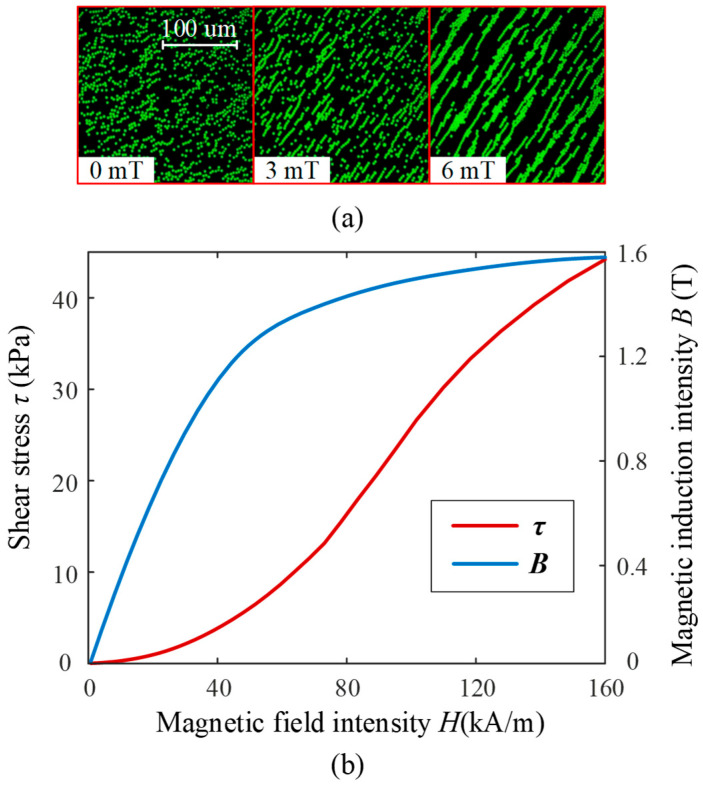
(**a**) Microstructure of MRF. (**b**) Shear stress and magnetization curves of MRF.

Due to the difficulty of achieving structural changes in mechanical systems during dynamic rotation, this paper adopts a magnetically controlled method to design a variable stiffness elastic element. MRF is a smart material controlled by spatial magnetic fields, characterized by high magnetic permeability and low hysteresis, primarily composed of magnetic particles, carrier fluid, and various surfactants. Under the influence of a magnetic field, MR fluid exhibits millisecond-scale reversible rheological effects. Using super-resolution fluorescence microscopy, dynamic changes in magnetic particles in water-based MR fluid (carbonyl iron powder, volume fraction 0.05%) are observed through an inverted microscope (Nikon ECLIPSE Ti) [32]. As shown in Figure 3a, without a magnetic field, the magnetic particles in MR fluid are evenly distributed, showing no magnetization, and the shear stress is minimal. When a magnetic field is applied, the magnetic particles align into chains along the field direction, exhibiting magnetization and resulting in some shear stress. As the magnetic field strength increases, the particle chains thicken, magnetization intensifies, and shear stress increases accordingly. Under a magnetic field, MRF can dynamically magnetize and alter shear stress, with the variation curve shown in Figure 3b.

**Figure 4:**
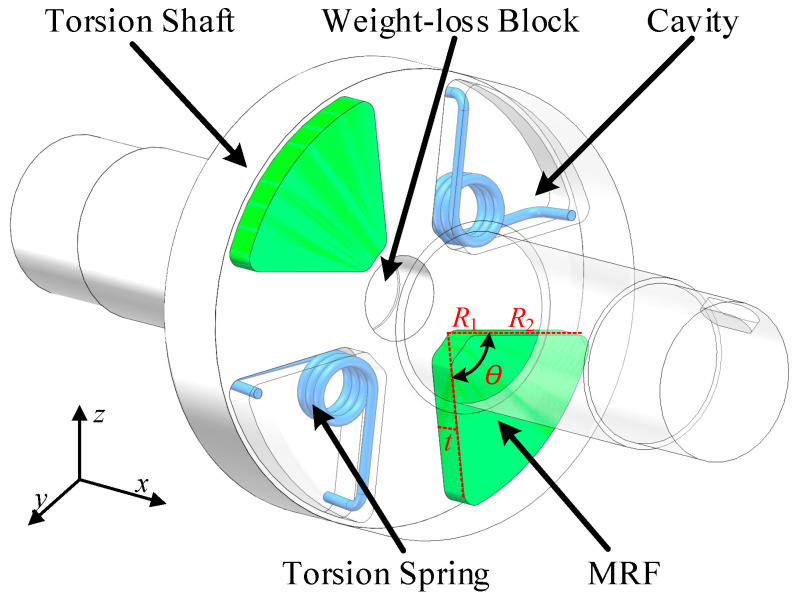
Three-dimensional model of the VSS.

Based on the high sensitivity of torsion springs and the adjustable stiffness capability of MR fluid, a magnetically controlled variable stiffness elastic element (VSS) is designed. Its main components include MR fluid, torsion springs, a cavity, and a torque shaft, as shown in Figure 4. Incorporating a suitable amount of torsion springs into the VSS ensures torque transmission without loss while increasing the maximum range of the VSS.

The VSS undergoes three main stages before and after torque application. In the first stage, without applied torque and in the absence of a magnetic field, the magnetic particles are evenly distributed, and the VSS is in a state of minimal stiffness. In the second stage, after applying an unknown torque, the magnetic field in the *z*-axis direction is adjusted to maximum. The MRF inside the magnetic control unit instantly magnetizes along the magnetic field direction, placing the VSS in a state of maximum stiffness, with minimal angular difference due to the torque. In the third stage, after obtaining a low-precision torque measurement, the magnetic field intensity in the *z*-axis direction is reduced, adjusting the VSS stiffness to an appropriate state. This increases the angular difference, further improving measurement accuracy.

## 3. Modeling of the VSS

### 3.1. Stiffness Modeling

The key to significantly adjusting the resolution and range of the sensor lies in the stiffness design of the VSS. The stiffness of the VSS comprises two components: the stiffness of the MRF and the stiffness of the torsion spring. The stiffness of the MRF determines the variable stiffness capability, while the stiffness of the torsion spring primarily determines the upper and lower limits of the range.

**Figure 5:**
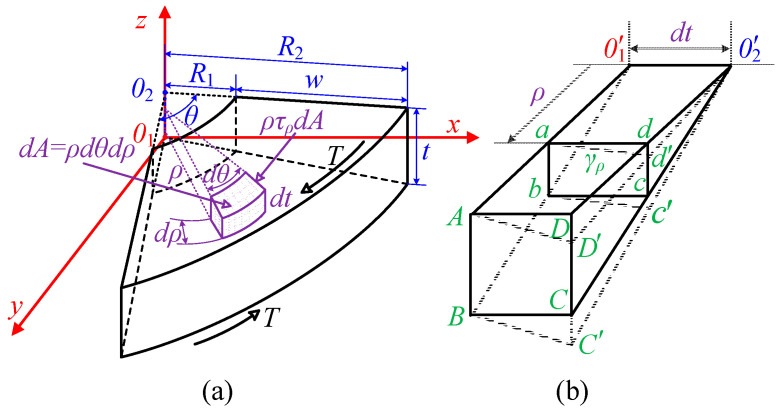
Physical model of MRF. (**a**) Force model; (**b**) equivalent model.

According to the small deformation theory in material mechanics, the cross-section of an elastic object remains planar during torsional deformation, with its shape and size unchanged. Therefore, by analyzing the internal torque of the MRF and deriving the polar moment of inertia of the cross-section, the relationship between torque and stiffness can be established. Torque *T* is applied along the *z*-axis to the MRF to cause torsional deformation, as shown in Figure 5a. The cross-section of the MRF in the direction of the torque is a sector ring. Here, *t* is the width, *R*_1_ is the inner diameter, *R*_2_ is the outer diameter, and *w* is the thickness.

At a micro-element *dA* located at a distance ρ from the center, there is a micro-shear force *ρτ_ρ_dA*. Across the entire cross-section, the torque equals the sum of all micro-shear forces:

(1) $$T=\int_{A}\rho \tau_{\rho }dA$$

To determine the stress *τ_ρ_* at each point on the cross-section, the deformation at each point within the MRF needs to be understood. For this, we analyze two cross-sections at a distance *dt* and an infinitesimally small wedge body; the deformation of the wedge is shown in dashed lines, as illustrated in Figure 5b. Any rectangle *abcd* located *ρ* from the center of the MRF deforms into a parallelogram *abc’d’* under shear stress. The relative angle between the two cross-sections of the wedge body is the torsion angle *dθ*; hence, the shear stress *τ_ρ_* of the rectangle *abcd* is:

(2) $$\tau_{\rho }=G\rho \frac{d\theta }{dt}$$

where *G* is the shear modulus of the MRF, and the direction of *τ_ρ_* is perpendicular to the radius. From the above formula, it can be seen that the torsional shear stress of the MRF varies linearly with the radius. Substituting the above formula into (1), the relationship between torque and shear modulus can be derived:

(3) $$T=\int_{A}\;G\rho^{2}\frac{d\theta }{dt}\cdot dA$$

Since the area of the micro-element is *dA* = *rdθdρ*, the polar moment of inertia *I_t_* of the MRF cross-section can be derived as:

(4) $$It=\int_{A}\rho^{2}dA=\int_{R_{1}}^{R_{2}}\int_{0}^{\theta }\rho^{3}d\theta dr=\frac{1}{4}\theta ({R_{2}}^{4}-{R_{1}}^{4})$$

Substituting the above formula into (3) and integrating over the width, the relationship between the stiffness *K*_1_ of the MRF and the torque can be obtained:

(5) $$K_{1}=\frac{T}{\theta }=\frac{G\theta ({R_{2}}^{4}-{R_{1}}^{4})}{4t}$$

The other component of VSS stiffness is the stiffness *K*_2_ of the torsion spring. Within the range of elastic deformation, the torsion spring has a proportional relationship between torque and the torsion angle. The expression for its stiffness *K*_2_ is derived as follows:

(6) $$K_{2}=4Ed^{4}/1167\pi D_{m}NR=4EI/\pi D_{m}N=T/\theta$$

where *K*_2_, *E*, *D_m_*, *N*, *R*, and *I* are the spring stiffness, modulus, pitch diameter, number of working circles, force arm, and inertia moment of the torsion spring, respectively, and *θ* is the torsion angle. Assuming the number of MRFs is *N*_1_ and the number of torsion springs is *N*_2_, the overall stiffness *K* of the VSS is a function of *G*, *N*_1_, *N*_2_, *t*, and *w*, which can be written as:

(7) $$K=\frac{N_{1}G\theta ({R_{2}}^{4}-{R_{1}}^{4})}{4t}+\frac{4N_{2}EI}{\pi D_{m}N}$$

From the stiffness (7) of the VSS, it can be seen that the variable stiffness performance of the VSS is directly proportional to the varying shear modulus and thickness of the MRF and inversely proportional to the width. The torsion spring can effectively enhance the range of the VSS. Since the measurable torque range depends on the maximum stiffness Kh, and the resolution depends on K, modifying these parameters allows us to expand and reduce the range.

### 3.2. Stiffness Simulation

**Figure 6:**
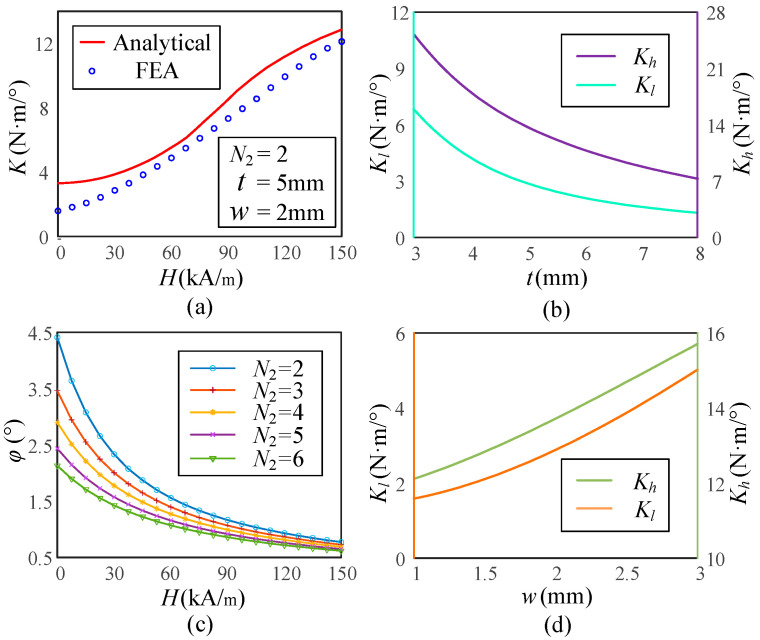
Simulated stiffness characteristics of the VSS. (**a**) Curves of stiffness versus magnetic field. (**b**) Influence of *t* on *K_h_* and *K_L_*. (**c**) Influence of *N*_2_ on the twist angle. (**d**) Influence of *w* on *K_h_* and *K_L_*.

The variable stiffness performance of the VSS with respect to the magnetic field strength and its geometric parameters is investigated using finite element analysis (FEA), as shown in Figure 6. In the investigation of magnetic field strength, the number of MRFs *N*_1_ and the number of torsion springs *N*_2_ are set to be 2, and the thickness *t* of 5 mm and the width *w* of 2 mm, and this setup is validated, as shown in Figure 6a. It can be observed that by controlling the magnetic field strength, the stiffness *K* of the VSS can be continuously adjusted over a wide range. Compared with theoretical analysis results, the FEA validation showed larger deviations but better linearity, primarily due to the linear modeling of the magnetorheological fluid. Non-linear modeling can improve the accuracy of FEA simulations and provide insights into the impact of geometric parameters on VSS stiffness. As shown in Figure 6b, as the thickness *t* increases, both the maximum stiffness *K_h_* and the minimum stiffness *K_l_* decrease, while the stiffness variation rate also decreases, indicating that the variable stiffness capability of the VSS is inversely proportional to *t*. Therefore, a smaller *t* should be selected in the design process. Applying a torque of 10 N·m, the effect of different *N*_2_ on the torsion angle *φ* is observed, as shown in Figure 6c. It is observed that in a weak magnetic field, the torsion angle *φ* decreases with increasing *N*_2_, with the reduction rate being relatively sensitive. In a moderate to strong magnetic field, the torsion angle *φ* decreases with increasing *N*_2_, but the reduction rate is less significant. This is mainly because, in a weak magnetic field, the shear stress of the MRF is too small, making the torsion spring have a greater impact on the stiffness. As the magnetic field strengthens, the influence of the torsion spring and the MRF on stiffness tends to become equal. As shown in Figure 6d, as the width *w* increases, *K_h_* and *K_l_* also increase. However, the stiffness variation rate remains almost unchanged, making this parameter helpful for better selecting the stiffness range. Therefore, the geometric parameters should be properly adjusted in the design to achieve the required stiffness variation without affecting other characteristics of the VSS.

## 4. Finite Element Analysis

FEA is an effective tool for analyzing the feasibility of mechanical equipment. First, the physical model unit is developed using commercial software SolidWorks 2024. Then, a simulation analysis of the VSS is conducted using ANSYS 2021 software to verify its mechanical performance and model accuracy. Steel wire for piano, grade B (SWP-B), is selected as the torsion spring material because of its good linear elastic behavior, high yield strength, and overall favorable mechanical properties. The material parameters and geometry parameters of the VSS are shown in Table 1.

**Figure 7:**
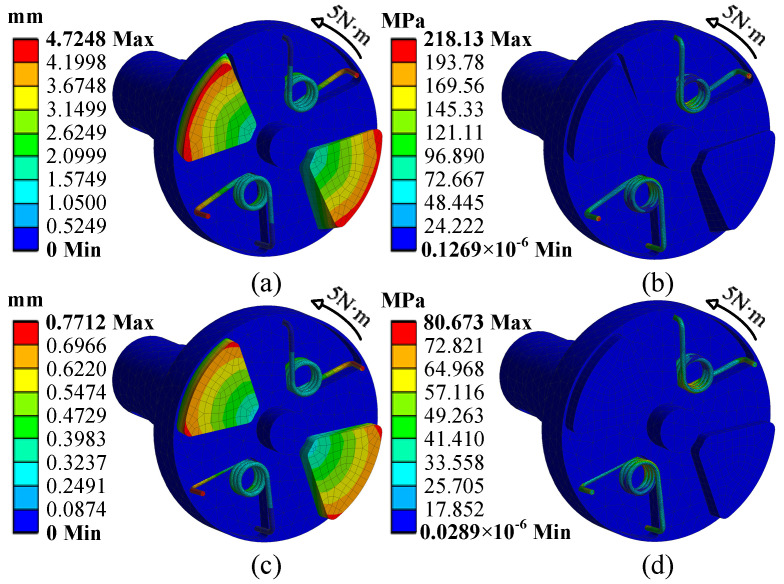
Deformation and stress distribution of the VSS. (**a**,**b**) The low stiffness state. (**c**,**d**) The high stiffness state.

First, a static simulation is performed on the VSS to evaluate its mechanical behavior in equilibrium. The left shaft of the VSS is subjected to fixed constraints, and the right shaft is set as a rigid body with a fixed torque of 5 N·m. A tetrahedral mesh is applied for discretization. The deformation and stress distribution in both the minimum and maximum stiffness states are simulated. The torsion angle is calculated from the deformation and spatial coordinates of the fixed points, as shown in Figure 7. Figure 7a,b depict the minimum stiffness state, while Figure 7c,d depict the maximum stiffness state. Notably, under the same applied torque, the torsion angles in the two states differ significantly. The torsion angle in the minimum stiffness state is 6.5667°, while in the maximum stiffness state, it is only 0.82264°. Therefore, the stiffness variation ratio of the VSS is 7.98.

Next, a modal analysis is conducted on the VSS. The results show that the first natural frequency of the VSS is 80.774 Hz in the minimum stiffness state and 152.691 Hz in the maximum stiffness state. The corresponding resonance speeds v are calculated as 4846.44 r/min and 9161.46 r/min. Additionally, to ensure the longevity of the torsion spring, the stress when the torsion spring reaches its maximum torsion angle should be controlled within 80% of the material’s yield stress. The simulation results fully meet this condition. Notably, under the same applied torque, the maximum stress in the minimum stiffness state is 218.12 MPa, while in the maximum stiffness state, it is only 80.673 MPa. Therefore, this further verifies that altering the shear stress of the magnetorheological fluid enables the range variation. In both states, the stress increases with the radius, which is consistent with the results derived from (2).

## 5. Angle Measurement Method Based on Screw Modulation

When subjected to torque, the VSS undergoes torsional deformation, resulting in an angular difference Δ*θ* between the two ends of the VSS shaft. With the stiffness of the VSS held constant, a linear relationship can be established between the angular difference, stiffness, and torque. Therefore, accurately measuring the angular difference enables the precise determination of torque.

**Figure 8:**
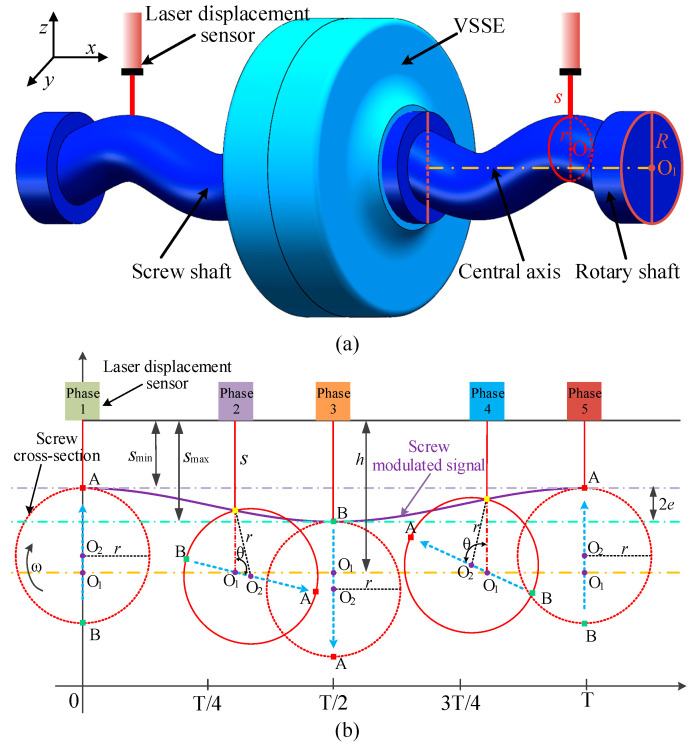
Principle of angle measurement based on screw modulation. (**a**) Screw modulation model. (**b**) Modulation principle.

This paper proposes a novel angle measurement method based on screw modulation. The mechanical structure of this method primarily consists of two sinusoidal screw shafts and two laser displacement sensors, as shown in Figure 8a. The rotation axis has a center O_1_ with a radius *R*. The cross-section of the screw shaft along the *x*-axis is a circle with center O_2_ and radius *r*. The screw shaft is formed by O_2_ performing periodic sinusoidal motion around the central axis and rotating about it. Since the center of mass of each cross-section of the screw shaft is located at O_2_, all these centers of mass collectively form a sinusoidal function. Since the integral of the sine function over one period equals zero, it can be concluded that the screw’s eccentricity relative to the central axis is zero. As a result, the screw shaft avoids eccentric inertia.

As the rotation axis rotates at a constant speed, the distance *s* between the laser displacement sensor and the circular cross-section of the screw varies with the angle. Figure 8b illustrates the five angular variations over one period. The output of the laser displacement sensor is a periodic quasi-sinusoidal signal with a high signal-to-noise ratio (SNR), known as the screw modulation signal. The distance between the laser displacement sensor and the central axis is *h*. To improve measurement accuracy, 2*e* should be maximized, so the laser displacement sensor should be aligned with the deepest or highest points of the screw, and *r* must be greater than half of *R*. Except for the unknown angle *θ*, all other parameters are known. Therefore, through simple trigonometric relationships, the screw modulation signal can be derived:

(8) $$\left\{\begin{array}{l} \theta =\arccos\left(\frac{{\left(h-s\right)}^{2}+R^{2}-2Rr}{2(h-s)(R-r)}\right)\\ h=s_{\min}+R\\ s_{\max}-s_{\min}=2e\\ \frac{R}{2}<r<R \end{array}\right.$$

(9) $$T=K_{\left(n\right)}\left(\left(\theta_{1}-\theta_{2}\right)-\left(\varphi_{1}-\varphi_{2}\right)\right)$$

The screw modulation-based angle measurement method does not require full-cycle sampling and can achieve high-precision instantaneous angle measurement. This method measures the angles *θ*_1_ and *θ*_2_ at both ends of the VSS and records the initial angles *φ*_1_ and *φ*_2_. Consequently, the torque measurement is obtained, as shown in (9). Due to the sinusoidal variation in the screw’s helical path along the axial direction, by decoupling the radial and axial screw modulation signals, this method enables the measurement of both instantaneous angles and axial displacement. Therefore, it is also applicable to the design of other sensors, such as force sensors.

## 6. Prototype Perseverance Tests

### 6.1. Prototype Development

**Figure 9:**
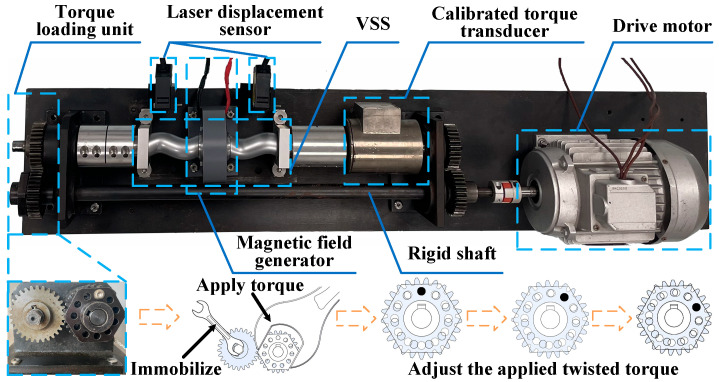
Power-closed torque test bench.

All mechanical components of the VSS are precisely machined and assembled. The VSSE has an outer diameter of 35 mm, an overall thickness of 27 mm, and an internal cavity radius of 16.3 mm. The screw shaft has a radius *r* of 10 mm and a shaft length of 63 mm, while the rotation shaft radius *R* is 14 mm. The complete sensor measures 153 mm in length and 70 mm in overall height. The screw is designed with a hollow structure and is connected to the solid shaft of the VSS via a keyway. This design ensures that the screw itself does not bear torque when the sensor is subjected to it. Thus, it prevents deformation due to torque, thereby eliminating this measurement error. A power-closed torque test bench is designed in this study for the calibration and performance evaluation of the VSS. The test bench is mainly composed of the VSS, two laser displacement sensors, a dynamic torque loading device, a reference torque sensor, a magnetic field generator, a drive motor, bearing blocks, and other connecting parts, as shown in Figure 9. The model of the laser displacement sensor is HL-G105-S-J, which achieves an accuracy of 1.5 µm within its measurement range and supports a minimum sampling interval of 200 µs. From (10), this corresponds to an angular measurement resolution of approximately 1.16′, indicating good temporal response and measurement precision. The voltage output signal from the laser displacement sensor is acquired using an NI-9234 data acquisition card with 24-bit resolution. The coil in the magnetic field generator has 400 turns, and the current *I* ranges from 0 to 5 A. When the current reaches 5 A, it is sufficient to generate the maximum magnetic field required by the MRF.

Dynamic torque loading is one of the key functions of the test bench. To achieve stable dynamic small torque loading while minimizing power loss, a power-closed torque loading unit is designed in this study. This unit consists of a loading gear and an adjustment sleeve. The loading gear is in a clearance fit with the loading shaft to facilitate load adjustment. The adjustment sleeve is connected to the loading shaft via a key, forming a closed torque loop. The loading gear has 12 evenly distributed holes, while the adjustment sleeve has 11. The loading mechanism is similar to the micrometer measurement principle. During loading, the appropriate matching hole positions on the gear and sleeve are selected based on the required torque value and then secured with bolts, achieving stable dynamic small torque loading. Additionally, the test bench is equipped with a 24-bit high-precision torque sensor as a reference, which is strain gauge-based and expensive, to achieve dynamic calibration of the VSS. During loading, the bolt shaft and hole wall have no clearance, and the bolt shaft is subjected to shear forces; therefore, an M8 hexagonal reamed bolt is used.

### 6.2. Prototype Evaluation

**Figure 10:**
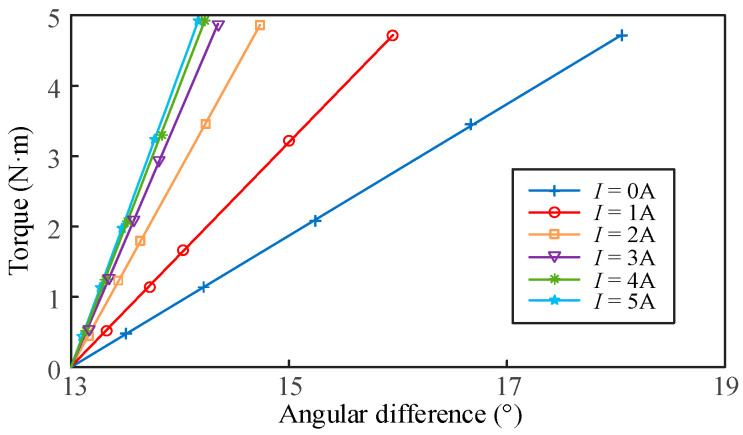
VSS calibration curves at different stiffnesses.

For the performance evaluation of the sensor, the first step is to calibrate the VSS under different stiffness conditions to obtain the linear relationship between the output angle difference and the applied torque. The output angle difference is measured using a laser displacement sensor and the screw shaft. Before calibration, the initial angle difference is recorded. During calibration, the output current of the DC power supply is adjusted in 1 A increments within the range of 0–5 A. The torque is gradually adjusted using the torque loading unit and then measured with a reference torque sensor. The calibration results of the VSS under different stiffness conditions are shown in Figure 10. It can be observed that the VSS of different stiffnesses has good linearity. The dynamic continuous adjustment of different stiffnesses is realized. At *I* = 0 A, the VSS exhibits the lowest stiffness and highest resolution, with values of 0.9352 N·m/° and 0.0178 N·m, respectively. At *I* = 5 A, the VSS exhibits the highest stiffness and lowest resolution, with values of 4.221 N·m/° and 0.08 N·m, respectively. The resolution primarily depends on the accuracy of the laser displacement sensor. The stiffness variation rate of the VSS is 4.5135.

The current prototype exhibits a range-to-resolution ratio of approximately 1120. Although this ratio is modest compared to certain micro-scale torque sensors, those devices are primarily targeted at measuring extremely small torques within very limited ranges. The novelty of our sensor lies in its continuously adjustable resolution. Therefore, this sensor can reduce stiffness to enhance measurement accuracy or increase stiffness to extend the measurement range, meeting different requirements for measurement accuracy and range under various conditions. The lower measurement limit is constrained by mechanical friction/stiction and measurement noise, while the upper limit is determined by the yield strength of the torsion spring material and the maximum sustainable shear stress of the MRF under the available magnetic field. The 5–20 N·m measurement range of the prototype is particularly relevant for applications such as robotic joint torque sensing, auxiliary drivetrain components in electric vehicles, test benches for small motors and actuators, and precision machinery requiring dynamic torque feedback.

**Figure 11:**
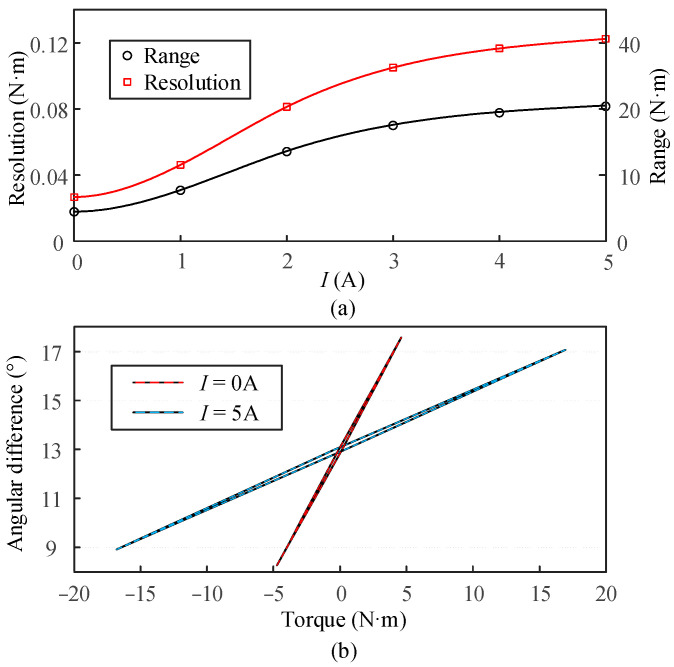
VSS sensor performance evaluation. (**a**) Resolution and range change under different stiffness. (**b**) Hysteresis characteristic evaluation.

Additionally, the range variation in the VSS under different stiffness conditions is also presented. The range is primarily measured based on the VSS’s ability to maintain good linearity and repeatability under maximum torque application. The resolution and range variation curves are well fitted using spline interpolation. The measurement results are shown in Figure 11a. It can be observed that the VSS can increase resolution by reducing the range or expand the range by lowering the resolution.

The hysteresis of the torque sensor depends on the maximum deviation obtained from cyclic loading and unloading tests. This study investigates the hysteresis characteristics of the VSS in its maximum and minimum stiffness states. The VSS is subjected to cyclic loading and unloading of torque up to approximately 80% of its range in both stiffness states, with the results shown in Figure 11b. The hysteresis is found to be 1.5% and 1.1% for the two states, respectively. Additionally, five sets of experiments are repeated to assess the repeatability of the VSS, resulting in values of 0.6% and 0.41%, respectively.

### 6.3. Validation of Dynamic Workflow

To verify the sensor workflow shown in Figure 2, we implemented a closed-loop control system to accomplish this process. This system performs the following steps algorithmically:

Firstly, when an unknown or varying torque is applied, the Xilinx AX7035 FPGA controls the current source driver to deliver the maximum current, thereby maintaining the sensor at maximum stiffness *K*_h_ for initial torque measurement. Laser displacement sensor signals are acquired via an NI-9234 DAQ module and transmitted to the FPGA through a serial interface, where the angular difference Δ*θ* is computed using Equation (8). Subsequently, the calibrated stiffness *K*_h_ and Equation (9) are employed to derive the preliminary torque estimate *T*_init_. Proceeding to the optimization phase, based on *T*_init_ and a predefined target resolution/range, the control algorithm dynamically calculates the optimal stiffness *K*. This step aims to maintain *T*_init_ at 80% of the new measurement range while maximizing resolution. Finally, the FPGA reacquires the laser displacement sensor signal to compute the final high-accuracy torque value *T*_meas_.

To evaluate the sensor’s dynamic performance, we added time response tests on top of the existing experiment to measure the latency of the full closed-loop workflow shown in Figure 2. Specifically, the response time was obtained from the FPGA counter and its crystal oscillator frequency. Counting started when the sensor began measuring the unknown torque and stopped when the sensor output the final measured torque *T*_meas_, yielding the elapsed measurement time *t*.

To validate this workflow experimentally, we used the torque loading unit on our test bench to apply five different unknown torque levels. The reference torque *T*_ref_ was measured by a high-precision calibrated torque sensor. Throughout the tests, *T*_init_, *T*_meas_, and *T*_ref_ were concurrently output for comparison, yielding the results presented in Table 2. Table 1 indicates that, while the sensor working at its maximum stiffness is able to span a wide range of roughly 20 N·m, the preliminary estimate *T*_init_ deviates noticeably from the reference torque *T*_ref_. Following stiffness adjustment for resolution maximization, the final torque output *T*_meas_ demonstrates close convergence to *T*_ref_.

The response times are accurate to 0.01 ms. All measured values are around 12 ms, demonstrating that the system is capable of completing a full measurement-update cycle within a short timeframe. This confirms that the proposed system is suitable for medium-speed dynamic torque sensing applications, such as robotic joint control, electric drivetrain calibration, or precision actuation, where torque variation bandwidths are typically below 50 Hz.

### 6.4. Future Work

Future work will focus on miniaturization. Potential improvements include optimizing the coil and magnetic circuit to reduce size, using higher-saturation magnetic materials to achieve the same stiffness change with a smaller volume of fluid, and integrating the torsion spring and fluid chamber into a single compact assembly. These changes aim to retain performance while approaching the compactness of conventional sensors. The relatively large physical dimensions of our current prototype primarily arise from the screw modulation mechanism employed for angle measurement. It is noteworthy that the proposed screw modulation method is not limited to our torque sensor alone but can also be applied broadly to other applications requiring high-precision angular measurements. Furthermore, if a further reduction in sensor size is desired in future designs, selecting compact, high-resolution angular sensors would be a promising alternative. In addition, we compared the proposed sensor with several representative torque sensors from the recent literature, as well as commercially available torque sensors, as summarized in Table 3. It can be observed from this comparative analysis that although our proposed sensor has somewhat higher structural complexity than conventional designs, it does not exhibit a significant disadvantage in terms of overall physical dimensions. The comparison also reveals that the sensor proposed in this work holds certain advantages in terms of minimum resolution and maximum measurement range. Furthermore, other existing torque sensors typically excel in high resolution at the expense of limited range, or vice versa. Consequently, our proposed sensor specifically addresses this inherent trade-off between resolution and measurement range. Due to current hardware limitations, our torque loading test bench supports only static torque levels through manual adjustment and thus cannot directly apply step or sinusoidal torque inputs. Future work will include upgrading the torque loading platform to enable programmable dynamic torque inputs for step response and frequency-domain performance analysis. We will conduct frequency response tests, step torque response measurements, and detection speed measurements on the sensor to better evaluate its overall performance.

## 7. Conclusions

This paper proposes a novel variable stiffness torque sensor. By integrating the advantages of torsion springs and MRF, the elastic element can achieve dynamic stiffness adjustment under varying magnetic field intensities, thus meeting the demands for resolution and range under different measurement conditions. The stiffness model of the VSS is established, and its theoretical accuracy is validated through finite element analysis. A screw modulation-based angle measurement method is proposed for the first time, achieving a theoretical angular resolution of 1.16′. A power-closed torque test bench is designed, and the VSS prototype is calibrated and performance-tested. The experimental results indicate that the sensor exhibits excellent linearity across different stiffness levels. Dynamic and continuous adjustment of resolution and range is achieved. At the lowest stiffness, the stiffness and resolution are 0.9352 N·m/° and 0.0178 N·m, respectively. At the highest stiffness, the stiffness and resolution are 4.221 N·m/° and 0.08 N·m, respectively. Furthermore, the sensor demonstrates favorable hysteresis characteristics and repeatability. Finally, closed-loop workflow experiments confirm the sensor’s effectiveness in measuring unknown torque.

## Figures and Tables

**Table 1:** The material parameters and geometry parameters of the VSS.

Parameters	Value	Unit
*N*_2_	2	-
*t*	5	mm
*w*	2	mm
τ	50	KPa
*R*_1_	9.3	mm
*R*_2_	25	mm
*E*	4.5	GPa

**Table 2:** Closed-loop dynamic workflow validation results.

Test	*T*_ref_ (N·m)	*T*_init_ (N·m)	*T*_meas_ (N·m)	*t* (ms)
1	0.6409	0.5819	0.6348	12.13
2	1.8515	1.7926	1.8225	12.13
3	3.2088	3.3067	3.2564	12.13
4	4.9421	5.0455	5.0085	11.95
5	8.5063	8.4301	8.4507	11.66

**Table 3:** Comparison of torque sensor parameters.

Source of Torque Sensor	Maximum Outer Diameter	Length	Thickness	Resolution	Range
Reference [7]	85 mm	165 mm	-	0.00859 mV/N·m	8 N·m
Reference [12]	82 mm	-	20 mm	320 mN·m	40 N·m
Reference [30]	68 mm	-	10 mm	1.6 mN·m	2.3 N·m
Commercial sensors	147 mm	210 mm	-	300 mN·m	100 N·m
Proposed sensor	70 mm	135 mm	27 mm	17.8–80 mN·m	5–20 N·m

## Data Availability

The data presented in this study are available on reasonable request from the corresponding author due to confidentiality restrictions.
